# Clinical perspectives on hospitals’ role in the opioid epidemic

**DOI:** 10.1186/s12913-020-05390-4

**Published:** 2020-06-08

**Authors:** Olena Mazurenko, Barbara T. Andraka-Christou, Matthew J. Bair, Areeba Y. Kara, Christopher A. Harle

**Affiliations:** 1 Department of Health Policy and Management, Indiana University Richard M. Fairbanks School of Public Health, 1050 Wishard Blvd, RG5135, Indianapolis, IN 46202 USA; 2 Department of Health Management & Informatics, University of Central Florida, Orlando, USA; 3 VA Center for Health Information and Communication, Indianapolis, USA; 4 Division of General Internal Medicine, Indiana University School of Medicine, Indianapolis, USA; 5 Regenstrief Institute, Inc., Indianapolis, USA; 6 Division of Clinical Medicine, Indiana University School of Medicine, Indianapolis, USA; 7 Department of Health Outcomes and Biomedical Informatics; College of Medicine, University of Florida, Gainesville, USA

**Keywords:** Hospital, Opioid epidemic, Perceptions, Clinician

## Abstract

**Background:**

Policymakers, legislators, and clinicians have raised concerns that hospital-based clinicians may be incentivized to inappropriately prescribe and administer opioids when addressing pain care needs of their patients, thus potentially contributing to the ongoing opioid epidemic in the United States. Given the need to involve all healthcare settings, including hospitals, in joint efforts to curb the opioid epidemic, it is essential to understand if clinicians perceive hospitals as contributors to the problem. Therefore, we examined clinical perspectives on the role of hospitals in the opioid epidemic.

**Methods:**

We conducted individual semi-structured interviews with 23 clinicians from 6 different acute care hospitals that are part of a single healthcare system in the Midwestern United States. Our participants were hospitalists (*N* = 12), inpatient registered nurses (*N* = 9), and inpatient adult nurse practitioners (*N* = 2). In the interviews, we asked clinicians whether hospitals play a role in the opioid epidemic, and if so, how hospitals may contribute to the epidemic. We used a qualitative thematic analysis approach to analyze coded text for patterns and themes and examined potential differences in themes by respondent type using Dedoose software.

**Results:**

The majority of clinicians believed hospitals contribute to the opioid epidemic. Multiple clinicians cited Center for Medicare and Medicaid Services’ (CMS) reimbursement policy and the Joint Commission’s report as drivers of inappropriate opioid prescribing in hospitals. Furthermore, numerous clinicians stated that opioids are inappropriately administered in the emergency department (ED), potentially as a mechanism to facilitate discharge and prevent re-admission. Many clinicians also described how overreliance on pre-populated pain care orders for surgical (orthopedic) patients, may be contributing to inappropriate opioid use in the hospital. Finally, clinicians suggested the following initiatives for hospitals to help address the crisis: 1) educating patients about negative consequences of using opioids long-term and setting realistic pain expectations; 2) educating medical staff about appropriate opioid prescribing practices, particularly for patients with complex chronic conditions (chronic pain; opioid use disorder (OUD)); and 3) strengthening the hospital leadership efforts to decrease inappropriate opioid use.

**Conclusions:**

Our findings can inform efforts at decreasing inappropriate opioid use in hospitals.

## Background

The United States (US) is in the midst of an opioid epidemic [1, 2]. More than 2 million Americans have an opioid use disorder (OUD) and 12 million report misusing opioids [1, 2]. Current efforts to curb the opioid epidemic are mainly focusing on how opioids are prescribed in the outpatient setting [3]. Given that opioids are used in 50 to 80% of 35 million inpatient stays annually, more attention is required to address opioid use in the hospital [4–7]. Importantly, patients who are exposed to opioids in the hospital are more likely to use opioids long-term (90+ days after discharge), increasing their risk for developing an OUD [4].

Policymakers, legislators, and clinicians have raised concerns that hospital-based clinicians may be incentivized to inappropriately prescribe opioids when addressing patients’ pain care needs [8]. Evidence suggests that clinicians may still rely on outdated Joint Commission standards that defined pain as a “*fifth vital sign*,” leading to overaggressive pain management during hospitalization [8]. Furthermore, clinicians may inappropriately order opioids due to pressures to obtain better patient care experience scores. Until 2018, the Centers for Medicare and Medicaid Services (CMS) used patient experiences with pain care as one of the criteria in determining hospital reimbursement. Indeed, hospitalists (hospital-based physicians) believe that pressures to obtain high patient care experience scores promote inappropriate opioid use in the hospital [9, 10]. Thus, it is plausible to assume that hospital-based clinicians may perceive that hospitals are playing a role in opioid epidemic due to complexity of inpatient opioid prescribing.

However, it is unknown if hospital-based clinicians see hospitals as contributing to the opioid epidemic, and if so, how. Given the need to involve all healthcare settings in efforts to curb the opioid epidemic, it is essential to understand if clinicians perceive hospitals as contributors to the problem. If they do, it is necessary to know what aspects of the hospital operation are seen as contributors to the opioid epidemic. If they do not, then they may be less likely to propose or adopt initiatives to decrease inappropriate opioid prescribing in hospitals. Additionally, clinicians can identify at-risk patient populations who may require additional resources to manage their pain appropriately in the hospital. The goal of this study was to gather clinician perspectives on the hospital’s role in the opioid epidemic, as well as perspectives on what hospital services and patient populations are associated with inappropriate opioid prescribing. We also sought clinician perspectives on how hospitals can help address the opioid epidemic. Findings from our study can inform practitioners and administrators interested in developing interventions to decrease inappropriate opioid prescribing in hospitals.

## Methods

We applied the consolidated criteria for reporting qualitative research (COREQ) checklist (See Additional file 1_COREQ checklist) for our qualitative study [11].

### Study participants

Our participants were recruited from 6 acute care hospitals that are part of one healthcare system in the Midwestern U.S. The healthcare system has almost 3000 beds throughout the state with 115,350 admissions per year. Our participants were full-time hospitalists, inpatient adult nurse practitioners, and inpatient registered nurses employed by the healthcare system. We used purposive sampling to achieve a distribution of clinicians who varied in terms of gender, race/ethnicity, years in practice, and hospital location [12]. We recruited participants directly providing pain care, such as by ordering and administering opioids, to hospitalized patients. Our additional inclusion criteria for the study participants were:1) full-time employment within the health system, and 2) an active medical license.

As described in our prior work [13], we employed three approaches to recruit study participants. First, per our request, the president of the medical staff at one of the hospitals sent e-mails with description of the study to over 90 hospitalists employed by the health system. Second, we asked five directors of clinical operations and chief nursing officers to introduce the study via e-mail to the inpatient unit managers and registered nurses throughout the health system. Third, we asked a senior system-level health care manager to share the study description electronically with the health system’s executive team. Interested participants connected with the team via e-mail to set up the time for an interview. We obtained verbal informed consent from each participant before the interview. We rewarded participants with a $50 gift card for their time. The Institutional Review Board (IRB) at our institution approved the study. The IRB approved using verbal consent due to nature of our study presenting no more than minimal risks of harm to participants. Per IRB recommendation, we ensured that each participant was given an opportunity to ask questions and received a copy of the information sheet describing the goals and procedures of our study.

### Data collection

We conducted semi-structured, in-person interviews between September 2017 and August 2019 using an interview guide developed based on a literature review and our research questions (See Additional file 2_Interview Guides). During interviews, we asked participants two broad questions: 1) "Do you think that hospitals are contributing to the opioid epidemic? (Explain your answer); 2) If the participants answered yes to the first question, then we asked: “What can hospitals do to help address the opioid epidemic?” Our interviews also probed participants on other topics (e.g., how patient care experience measures affect clinician decision-making) that are presented elsewhere [13]. These interviews were conducted as part of a larger project examining the association between opioid prescribing and patient care experiences of hospitalized patient. In this paper, we describe themes that emerged from a fraction of the interview. We audio-recorded each interview with verbal consent, and transcribed it for analysis. Each interview lasted approximately 45 min. We asked two hospitalists and one nurse to pilot test the interview guide for comprehension and content prior to the start of the data collection. At the beginning of the interview every participant reported demographic and employment history.

### Analysis

Two researchers [OM, BA] performed a qualitative thematic analysis of the interview transcripts [14]. First, after the initial screening of several transcripts and review of the research questions a preliminary codebook was created. Researchers tested whether the codebook was reliable by individually applying preliminary codes to two transcripts. Next, the researchers assessed the accuracy and consistency of the preliminary codebook and subsequently adjusted it. They then coded each transcript individually using the revised codebook, followed by consensus coding of each transcript. As researchers were going through consensus coding process, they identified and added new codes to the codebook. The researchers consistently applied the final codes to all transcripts [15]. Next, researchers analyzed the coded text for patterns and themes and examined whether emerging themes were different depending on the respondent type [16]. Finally, the researchers identified overarching themes with representative quotes. The process of coding transcripts and reviewing coded data for themes occurred concurrently with data collection in an iterative process. We used Dedoose version 8.2.14 (Los Angeles, California) qualitative analysis software for coding and analysis.

Participant recruitment, interviews, and data analysis continued until no new codes emerged, and thematic saturation was achieved. In line with prior literature, we defined thematic saturation as a point at which collecting further interviews would not yield new information related to our research questions. Thus, we concluded that we reached thematic saturation when we repeatedly observed similar instances of data and our themes were well developed [17]. In other words, we stopped conducting interviews when we realized that we would have diminishing returns of conceptual or thematic depth from conducting additional interviews. All team members participated in developing the manuscript.

We followed established qualitative methodology procedures throughout our data analysis to ensure that our findings are rigorous and valid. We continually questioned how we interpreted the data and documented potential preconceptions and biases. We also proactively looked for the depth of the theme’s description (e.g., rich expressions of the issue by participants), and sought out alternative explanations of the data [18–20].

## Results

### Participant characteristics

Of the 23 participants, 12 were hospitalists, 2 were inpatient adult nurse practitioners, and 9 were inpatient registered nurses employed at six different acute care hospitals. We present detailed information about study participants in Table 1. Briefly, approximately half of the participants were females (14/23). Two-thirds of the participants were White (17/23). Our participants had, on average, 11 years of clinical experience. Our participants were representative of hospital-based clinicians nationally [21, 22]. We present the major themes with illustrative quotes below (See Table 2 for additional quotes). We did not observe unique response patterns across themes based on participant characteristics or hospital location.

**Table 1:** Participant characteristics (*N* = 23)

	**Hospitalist (*n*** **= 12)**	**Registered nurse (*n*** **= 9)**	**Adult nurse practitioner (*n*** **= 2)**	**Total**
Female, n (%)	3 (21)	9 (65)	2 (14)	14 (61)
Race/ethnicity, n (%)				
White	7 (41)	8 (47)	2 (12)	17 (74)
African-American	0	1 (100)	0	1 (4.3)
Asian	5 (100)	0	0	5 (21.7)
Work experience (years)*				
Mean	12	11	25	
Range	2–18	1–32	10–40	n/a
Years with an organization				
Mean	7.1	5.2	14	
Range	1–18	1–15	8–20	n/a
Hospital locations				
Hospital #1	3 (60)	0	2 (40)	5 (21.7)
Hospital #2	3 (50)	3 (50)	0	6 (26.2)
Hospital #3	2 (40)	3 (60)	0	5 (21.7)
Hospital #4	2 (100)	0	0	2 (8.7)
Hospital #5	1 (33.4)	2 (66.6)	0	3 (13)
Hospital #6	1 (50)	1 (50)	0	2 (8.7)

Note: Work experience excludes time in training, such as residency; Hospitalist- a physician with primary professional focus is the general medical care of hospitalized patients

**Table 2:** Emergent Themes with Illustrative Quotes

**Theme**	**Illustrative Quote**
**Hospital contributes to the opioid epidemic**	I think definitely inpatient medicine has a significant role. I think it starts in the emergency department. You know, it’s, God love em, cause I want to do that ER job, but you know folks come in and they are hurting. Once again, if they are truly hurting and you are treating them appropriately, I think it is okay. I think, however, if you are kind of coming in and maybe there is secondary gain and you are getting big doses of a strong narcotic, then you realize, well I don’t have to buy it off the street, I will just go the ER, which we see those patients that are going around (Hospitalist, #2).
	Hospital plays a huge role. You see somebody for IV drug use and say, “How did you get started?” “Oh, I had surgery. I was prescribed oxycodone. I started to get a number of refills, next thing you know, I was buying heroin on the street.” And so I think pills are a starting point. So I think the hospital plays a huge role (Hospitalist, #11).
	I think they [hospitals] can be. For instance, when we were the previous hospital, we had certain doctors. The patients would call in and ask what doctor was on because they knew certain doctors would give them things, other doctors wouldn’t. I could definitely see how it could be a factor (Nurse, # 5).
Potential Mechanisms of Hospitals’ Contribution to the Opioid Epidemic	
• Concerns about CMS reimbursement policy tied to patient care experience and Joint Commission’s report	You have someone who... gastroparesis, and instead of following up with their primary care doctor, they just keep returning to the emergency room. For the sake of patient satisfaction scores, we give in and we just keep giving them pain medicine (Nurse #10)
• Opioids are inappropriately administered in emergency department (ED)	I believe that ED is the place that is contributing to the opioid epidemic, because they treat them and street them, you know, they have to get them out. They do not meet the criteria for admission, maybe they are self-pay, they have no insurance at all, so what can we do to get them out the door? (Nurse, #7)
	ED often gives patients very large doses of pain meds and we try to break the cycle, but often they end up having to go home on opioids. I think it is definitely part of the circle (Hospitalist, # 9)
	I think the emergency department really freely gives a lot of narcotics, especially even IV narcotics (Nurse practitioner, #2)
• Using opioids is a pragmatic tool to facilitate discharge and prevent re-admission	This is prescription for your 60 pills, don’t come back to the hospital within a month because we know that if a patient comes back within 30 days it’s like the re-admission and it’s, it’s for hospitalists, another hit. So we tend to give them a 30 day supply so that even if you run out the medication, it will be after 30 days. Even if you come back, it doesn’t affect hospital (Hospitalist, #3).
• Overreliance on pre-populated pain care orders for surgical (orthopedic) patients	Like if some people who go through elective surgeries... I mean, there are batches of people who are probably higher risk for addiction that others and those batches who end up going through elective surgery, whether it is justified or not justified, they get... they will end up leaving with an opioid, like for sure (Hospitalist, #8)
	And it is just I think the surgeon is like, here, you need to take this medicine when you’re done with surgery because it will help with the pain. Instead of educating them and talking to them (Nurse #6)
Patient Populations at Risk for Inappropriate Opioid Use	
• Using opioids for opioid-naïve patients, especially following a surgery	I know several personal friends that have you been in that situation. Who were given opioids, OxyContin or other stuff while they were in the hospital and then they would never be able to get off of that. (Nurse, #4)
	I have seen it where people come in for pain control and I get that, but when they have like high doses of Dilaudid every two hours as needed and then they get sent home the next day, like going from IV to whatever. I mean, patients talk about having the withdrawal from it (Nurse, #8).
	If some people who go through elective surgeries... I mean, there are batches of people who are probably higher risk for addiction than others and those patients who end up going through elective surgery, whether it is justified or not justified, they get... they will end up leaving with an opioid, like for sure. Then that is sort of the start of a cascade of their problems along the way (Hospitalist, #8).
**Hospital Initiatives for Addressing the Opioid Epidemic**	
• Educating patients about negative consequences of using opioids long term and setting realistic pain expectations	A lot of the patients that I say that very same thing to say, “Oh, well my primary doctor just gives it to me, so I thought it was fine.” I say, “Well, they have side effects and your body gets used to those, and they’re also not the best medication for chronic pain.” I do, I feel like a lot of people just haven’t been educated. They don’t understand things that go on, the bad side effects that go along with taking narcotics (Nurse Practitioner, #2)
	Some people are like they-- when we educate them, they understand. Okay, what’s the complication of these opioids? So then they prefer non-opioids. So they try to say can you give me some tramadol, ibuprofen, and that’s still despite that if they are in immense pain and then during the hospital course, it’s like how effectively we’ve been able to treat the pain. And they will be willing to do anything to get back to their normal life. So when we tell them with these opioids, you can have these side effects, and they will be oh, no. I don’t want opioids. I will just take this (Physician #7).
	Patient education is essential. On the risks of continued dependence that can lead to addiction, that can lead to such deleterious effects that can be deadly (Nurse #7)
• Educating medical staff about appropriate opioid prescribing practices, particularly for patients with complex chronic conditions (chronic pain; opioid use disorder);	I would start from med school. I would first start from teaching moralities to all the med students because they are going to be the coming physicians. And then educate nursing students too because they will be like frontline workers administering opioids. And then at the same time also, educating the medical field. Everybody has to understand what opioid tolerance is (Hospitalist, #7)
	Put in education both for public and providers to be able to say, this is not okay to be able to prescribe quantities or be on medicines indefinitely and so forth, that there is sort of an upper limit or you have to jump through higher and higher levels of thresholds to be able to, like there are drugs that I don’t prescribe as a doctor that are available to a cardiologist, because they have got some extra training and we want to limit it to a certain.... I don’t prescribe any chemotherapy. We limit that to the oncologists. They have the additional training (Physician #4)
• Having a hospital leadership	I think you have to have interest from the frontline technicians and you would definitely have to have support and alignment from leadership to make it happen (Hospitalist, #12)
	I think just putting things in the limelight and giving it attention, may change it. Narcotic stewardship, has never had the limelight. No one’s really cared. so I think understanding that it’s a problem and giving it resources and giving it the limelight will help (Hospitalist, #11)
	I think, you know, having some leadership into, hey, this is a problem to work on, and I don’t think it quite exists in the political structure of local, state, national (Hospitalist, #5)

### The hospital role in the opioid epidemic

The vast majority of clinicians believed hospitals are contributing to the opioid epidemic. Some clinicians stated that hospitals play a major role relative to other healthcare settings, whereas others indicated that every healthcare setting, including hospitals, are contributing to the opioid epidemic. The illustrative quotes below highlight these two somewhat divergent perspectives on the hospital role in the opioid epidemic:

*The hospital plays a huge role. You see somebody for IV drug use and say, "How did you get started?" "Oh, I had surgery. I was prescribed oxycodone. I started to get a number of refills, next thing you know, I was buying heroin on the street." And so I think pills are a starting point. So I think the hospital plays a huge role (Hospitalist, #11).*

*I think every place that provides care, not only hospitals, but outpatients, urgent cares, every ED, I think every part has contributed to the opiate crisis (Hospitalist, # 12)*

As an exception, two clinicians did not believe that hospitals are contributing to the opioid epidemic due to the short-term nature of opioid prescribing in the hospital.

### Mechanisms of hospital contribution to the opioid epidemic

Clinicians described several mechanisms by which hospitals contribute to the opioid epidemic and identified specific patient populations at risk of being prescribed opioids inappropriately.

#### CMS reimbursement policy tied to patient care experience

First, multiple clinicians mentioned the CMS reimbursement policy tied to patient care experience scores and a 2000 Joint Commission report of pain as the “*fifth vital sign*” as influencing inappropriate opioid use. As one hospitalist explained it:

*If you get sick, you know that a hospital does not get paid, like it is audited on whether your pain was well controlled. So, everybody tried to focus everything on patient satisfaction [patient care experience measure], we do not want to have "not satisfied" in the pain question. Another issue is that we always write this pain scale. And that created another problem. I think hospitals were forced to do that because it came out as such a big thing. It is like a vital sign. Pain should be controlled, doesn't matter what dose of pain medication we use, but that is not always the best thing to do (Hospitalist, # 2)*

#### Prescribing opioids in the emergency department

Second, numerous clinicians stated that opioids are inappropriately used in emergency departments (EDs) for several reasons. First, some clinicians believed that opioids are used as a mechanism to facilitate discharging patients from the hospital or avoiding re-admissions. As one registered nurse described it:

*I believe that ED is the place that is contributing to the opioid epidemic, because they treat them and street them, they have to get them out. They do not meet the criteria for admission, maybe they are self-pay, they have no insurance at all, so what can we do to get them out the door? (Nurse, # 7)*

Other clinicians described difficulties in differentiating between patients with “true pain” versus patients without pain seeking opioids for misuse. Clinicians believed it is challenging to distinguish between these two groups of patients due to a lack of established patient-clinician relationships and time constrains, preventing in-depth conversations to collect comprehensive medical history. Clinicians stated that these challenges might be particularly prominent in the ED, where a high volume of patients and time constraints amplify a sense of urgency in treating patients. Finally, a few clinicians referenced ED-specific patient care experience scores that may also influence an inappropriate use of opioids in the ED.

#### Overreliance on pre-populated pain care orders

Many clinicians also described how overreliance on pre-populated pain care orders (i.e., generic order sets) might contribute to inappropriate opioid prescribing in the hospital. Specifically, patients undergoing orthopedic surgeries with prolonged hospitalizations and complex pain care were identified as being at risk of receiving opioids long-term following hospitalization, and, therefore, have a higher likelihood of developing OUD. Several clinicians also discussed how physicians might not hesitate to prescribe high or long-term dosages of opioids to post-surgical orthopedic patients who often appear to have “real” pain following a procedure (as opposed to seeking opioids for misuse). Furthermore, our participants explained how lack of care coordination between the hospital and outpatient setting places patients at risk following surgery, especially if no outpatient plan exists to wean patients off opioids and to avoid withdrawal symptoms following hospitalization. An example below describes how a hospitalist perceives the negative consequences of using pre-populated pain care order sets:

*There is an order set for these [orthopedic] procedures. But these orders are very generic, and each person gets the same thing, so everybody who has had a knee replacement will get that same regimen of pain control, and one of them is an opioid. Is that really necessary? Or some patients say, I don't have pain, but they sent me home with narcotics (Hospitalist, # 9)*

#### Patient populations at risk for inappropriate opioid prescribing

Some clinicians identified specific patient populations at higher risk of receiving opioids inappropriately, which could then lead to developing or worsening OUD.

- Prescribing opioids to opioid-naïve patients

Participants described potential dangers of exposing opioid naïve patients, meaning those without a recent history of opioid use, to opioids in the hospital, with such patients sometimes then using opioids long-term and eventually developing OUD. As one nurse explained:

*I know several personal friends that have been in that situation. Who were given opioids, OxyContin or other stuff while they were in the hospital and then they would never be able to get off that. (Nurse, # 7)*

b. Prescribing opioids to patients with OUD

Participants believed that administering opioids in the hospital to individuals who are seeking drugs for misuse or who already have a confirmed diagnosis of OUD contributes to the opioid epidemic. Specifically, clinicians are often unable to distinguish between patients with “true pain” and patients who were seeking opioids for misuse. Furthermore, participants identified patients with OUD undergoing an elective surgery as at risk of increased health problems if the treating surgeon or hospitalist is unaware of a preexisting OUD. Due to underuse of the state’s prescription drug monitoring program (PDMP) (which was not mandatory at the time of the interviews), hospitalists and surgeons may be unaware of patients’ aberrant-drug use. As one hospitalist described the challenges associated with using PDMP:

*I would like to do it more. Because I think it is a clinical took that you can use, not just, hey you tell me you are not on anything but you've got 60. But, it is cumbersome. I have to remember my login, have to print out a face sheet or have a computer open to get their name and their address. It is just not a smooth system. It is good and get in on the PDF, but there is just so much involved in getting it, but when you do get it, it is there. But it is not like, you can't take it into the room and say, look at this (Hospitalist #2).*

### Hospital initiatives for addressing the opioid epidemic

Clinicians suggested several ways for hospitals to help address the opioid epidemic. First, multiple clinicians described the need to educate patients about the long-term risks of opioids and to set realistic pain expectations, thereby decreasing patients’ demands for opioids following hospitalization. As one inpatient nurse practitioner described it:

*I think as far as trying to curb it, I think it's going to be our job to more educate our patients. Especially about the dangers of narcotics, and especially about the fact that hey, narcotics long term are not good. Narcotics are not the best choice for chronic pain. (Nurse Practitioner, #1).*

Several clinicians noted the need to provide information about outpatient resources for managing pain beyond the hospital setting (e.g., pain specialists, physical therapy) that could help wean patients off opioids effectively and safely. A few clinicians also mentioned the need to provide patients with OUD with local treatment and support resources (e.g., rehabilitation centers, peer support groups). However, these clinicians often mentioned a lack of local resources for these complex patients. Additionally, they said that some patients would not contact the resources even if they were provided with information.

Second, some clinicians expressed the need to educate medical staff about appropriate opioid prescribing practices, particularly for patients with complex chronic pain conditions (e.g., chronic pancreatitis, fibromyalgia, etc.), OUD, and others. For example, one hospitalist described her ideas as follows:

*Online CMEs [continued medical education sessions], maybe lunch and learn sessions, those sorts of things would maybe keep these topics [appropriate opioid prescribing practices] fresh in our mind and keep us at the end of the things that you're supposed to do (Hospitalist, #12).*

A few clinicians also emphasized the importance of consistently using the PDMP to prevent inappropriate opioid prescribing, particularly for patients with OUD. Finally, some clinicians believed that it is essential to strengthening hospital leadership efforts to decrease improper opioid use. For instance, clinicians provided examples of antibiotic stewardship or infection prevention initiatives as a model (see the quote below):

*Some kind of hospital-wide initiative, like infection prevention, where they focus on those sorts of units, they take certain measures and they might give certain prizes to people that take those measures for these things. So possibly doing that in the same way as far as those other interventions, reaching out to the managers and units and having talks with some of the nurses and finding out how they think they would be able to improve on each individual unit, so that way you cover the hospital as a whole (Nurse, # 9).*

## Discussion

Our objective was to examine clinical perspectives on the hospital role in the opioid epidemic. We found that the majority of clinicians in our sample, regardless of the clinician’s role (physician, nurse, nurse practitioner), perceived that hospitals are contributing to the opioid epidemic. This finding is somewhat surprising given that clinicians often have different perceptions of hospital operations, including patient safety, working conditions, and leadership styles, based on their role [23, 24]. Importantly, such uniform agreement on the role of the hospitals in the opioid epidemic might signify high levels of awareness among clinicians regarding the opioid epidemic. Clinician awareness often serves as a critical step in changing how care is delivered [25, 26]. Thus, future studies should examine whether clinician awareness of hospitals’ contribution to the opioid crisis is associated with the successful implementation of hospital initiatives to decrease inappropriate opioid prescribing.

Clinicians, who believed that hospitals are contributing to the opioid epidemic cited CMS’s reimbursement policy tied to patient care experience measures, and the 2000 Joint Commission’s report, as potential drivers for inappropriate opioid administration in hospitals. Our findings are supportive of earlier qualitative research that documents how physicians’ concerns about patient care experience scores are influencing their decisions to prescribe opioids [9, 10, 27]. At the same time, evidence on the association between opioid prescribing and patient care experience in the hospital is mixed [28–30]. Nevertheless, CMS decided to temporarily remove two pain-care patient care experience questions from the hospital reimbursement formula starting in 2018 [31]. The CMS plans to add questions on communication about pain care instead. Future studies should examine whether these new pain care communication questions will alter opioid prescribing patterns.

Clinicians also stated several mechanisms by which opioids are inappropriately administered in the ED. For instance, some clinicians described opioids as a practical tool to facilitate discharge from the hospital and avoid re-admission. Our findings add to prior work [10] indicating that hospitalists prescribe opioids to facilitate discharges and prevent re-admissions. Our study suggests that inpatient clinicians perceive that ED clinicians may be using similar practices to avoid admitting patients and to facilitate discharges. Our findings highlight the multitude of competing pressures that hospital-based clinicians face, including the need to provide value-based care while balancing patient-centeredness and patient safety [13]. However, to date it is unknown whether opioid prescribing actually contributes to improved hospital efficiency and reduced hospital discharges.

Several clinicians perceived that using pre-populated order sets for pain care in patients undergoing surgeries in general, and orthopedic surgeries in particular, may be contributing to the opioid epidemic. Clinicians described how these pre-populated order sets are used without taking individual patient characteristics (e.g., age, history of prior exposure to opioids), and preferences into account. Importantly, the use of pre-populated order sets suggests the avoidance of fully considering patients’ values, goals, and preferences, and thus may be inconsistent with providing patient-centered care [32]. Given that patient-centered care is increasingly considered an essential aspect of health care, additional resources should be allocated to help clinicians use pre-populated order sets appropriately.

A few clinicians who believed that hospitals are contributing to the opioid epidemic also mentioned specific patient populations, such as opioid-naïve patients and patients with an OUD, who are at risk of inappropriately receiving opioids. Opioid-naive patients who receive opioids in the hospital are more likely to be on long-term opioid therapy compared to patients not exposed to opioids in the hospital and are subsequently at higher risk of developing OUD [33]. Future research should develop rigorous risk-based models to predict which inpatients should receive opioids and for how long. Some clinicians also described individuals with OUD as being at risk of inappropriately receiving opioids in the hospital. Experts raise concerns about the lack of guidelines for hospitalists regarding how to identify individuals with OUD and manage their pain in the hospital [34]. Our study suggests that in the ED, the ability to identify patients with OUD may be hampered by time constraints that prevent in-depth conversations with patients about their medical history. Thus, future studies should focus on developing and validating mechanisms for identifying individuals with OUD and guidelines to address their pain care needs in the hospital in general, and ED in particular.

Our participants also identified several strategies that hospitals can implement to help address the opioid epidemic. First, several clinicians mentioned the need to educate patients about the long-term risks of opioids and to set realistic pain expectations, thereby decreasing patients’ demands for opioids following hospitalization. Recent initiatives, including the 2016 Guideline on the Management of Postoperative Pain [35], stress the importance of educating patients on opioid tapering and use of analgesics after hospital discharge. Nevertheless, the evidence for the effectiveness of educational interventions for hospitalized patients on how to taper opioids and outpatient pain management following hospitalization is limited [36]. Importantly, studies show that educational interventions among patients with chronic pain are minimally effective [37]. Thus, patient educational interventions may need to be supplemented with additional institutional policies that decrease opioid prescribing. Second, several clinicians mentioned the need to educate clinicians on the appropriate use of opioids. Prior studies show that educating clinicians appears to be a minimally effective strategy, particularly for ones providing care in high-cognitive load environments, meaning environments that require completion of multiple mental tasks during the short period, such as acute care settings [38]. Therefore, additional tools are needed to support clinicians in making evidence-based decisions. Decision-support tools integrated with patients’ medical records could provide relevant, timely, and accurate patient information, potentially improving how clinicians order opioids to hospitalized patients in pain. Future studies should focus on the design and implementation of decision-support tools for pain care in the hospital setting.

Finally, a few clinicians stressed the importance of consistently checking the state PDMP to prevent inappropriate opioid prescribing for non-surgical inpatients. Briefly, the PDMP collects and tracks all controlled substances dispensed to the residents in our state. The PDMP also generates reports to all registered healthcare providers in the state on controlled substance prescriptions for a given patient. National and local studies found that mandatory PDMP use is associated with reductions in opioid prescribing rates in the outpatient setting [39–41]. Our state introduced mandatory PDMP utilization in the hospital relatively recently (January 1st, 2020). Thus, we lack evidence of the effectiveness of the state PDMP on opioid prescribing rates among non-surgical inpatients. Future studies should comprehensively examine the potential impact of state PDMPs on rates of inappropriate opioid prescribing among non-surgical inpatients.

### Limitations

Although our participants came from 6 different acute care hospitals, they were all part of a single healthcare system in a Midwestern state. Thus, our findings may not generalize to other hospital settings. Furthermore, selection bias of individuals who were willing and able to participate in the interviews may have influenced the discussions. On a similar note, although our participants were representative of hospital-based clinicians nationally in terms of race/ethnicity, a more racially/ethnically diverse sample may have yielded different findings. Finally, although we have sought perspectives of key stakeholders, such as physicians and nurses, a more diverse sample of participants could have elicited additional views.

## Conclusions

Hospital-based clinicians perceive that hospitals are contributing to the opioid epidemic. Opioid-naïve patients and patients with an OUD may be at particular risk of inappropriately receiving opioids. Our study suggests that the ED is a location of particular importance for administrators to introduce interventions to limit inappropriate opioid prescribing. Future studies should examine the effectiveness of various interventions proposed by clinicians in addressing inappropriate opioid administration in the hospital.

## Supplementary information

**Additional file 1.** The COREQ checklist by Tong, Sainsbury and Craig (2007) completed for the study: Clinical perspectives on hospitals’ role in the opioid epidemic.

**Additional file 2.** Clinicians Interview Guide.

## Data Availability

The datasets used and/or analyzed during the current study are available from the corresponding author on reasonable request.

## Abbreviations

CMC: Center for Medicare and Medicaid Services
COREQ: Consolidated criteria for reporting qualitative research
ED: Emergency department
IRB: Institutional Review Board
OUD: Opioid use disorder
PDMP: Prescription drug monitoring program
US: The United States.

## References

[1] Guy JG, Zhang K, Bohm MK, Losby J, Lewis B, Young R (2017). Vital signs: changes in opioid prescribing in the United States, 2006-2015. MMWR Morb Mortal Wkly Rep.

[2] Schuchat A, Houry D, Guy GP (2017). New data on opioid use and prescribing in the United States. JAMA.

[3] Center for Disease Control and Prevention (2016). CDC guideline for prescribing opioids for chronic pain—United States, 2016. Morb Mortal Wkly Rep.

[4] Donohue JM, Kennedy JN, Seymour CW, Girard TD, Lo-Ciganic W-H, Kim CH (2019). Patterns of opioid administration among opioid-naive inpatients and associations with Postdischarge opioid use: a cohort study. Ann Intern Med.

[5] Burden M, Keniston A, Wallace M, Busse J, Casademont J, Chadaga S (2019). Opioid utilization and perception of pain control in hospitalized patients: a cross-sectional study of 11 sites in 8 countries. J Hosp Med.

[6] Herzig SJ, Rothberg MB, Cheung M, Ngo LH, Marcantonio ER (2014). Opioid utilization and opioid-related adverse events in nonsurgical patients in US hospitals. J Hosp Med.

[7] HCUP Stats. Trends in Inpatient Stays 2019 [Available from: https://www.hcup-us.ahrq.gov/faststats/NationalTrendsServlet.

[8] Baker DW (2017). History of the joint Commission’s pain standards: lessons for today’s prescription opioid epidemic. JAMA.

[9] Zgierska A, Rabago D, Miller MM (2014). Impact of patient satisfaction ratings on physicians and clinical care. Patient Prefer Adherence.

[10] Calcaterra SL, Drabkin AD, Leslie SE, Doyle R, Koester S, Frank JW (2016). The hospitalist perspective on opioid prescribing: a qualitative analysis. J Hosp Med.

[11] Tong A, Sainsbury P, Craig J (2007). Consolidated criteria for reporting qualitative research (COREQ): a 32-item checklist for interviews and focus groups. Int J Qual Health Care.

[12] Green J, Thorogood N (2018). Qualitative methods for health research: sage.

[13] Mazurenko O, Andraka-Christou BT, Bair MJ, Kara AY, Harle CA. Balancing patient-centered and safe pain Care for Nonsurgical Inpatients: clinical and managerial perspectives. Jt Comm J Qual Patient Saf. 2018. 10.1016/j.jcjq.2018.11.004 PMC6491228 30591269

[14] Fereday J, Muir-Cochrane E (2006). Demonstrating rigor using thematic analysis: a hybrid approach of inductive and deductive coding and theme development. Int J Qual Methods.

[15] Bradley EH, Curry LA, Devers KJ (2007). Qualitative data analysis for health services research: developing taxonomy, themes, and theory. Health Serv Res.

[16] Crabtree BF, Miller WL (1999). Doing qualitative research: sage publications.

[17] Saunders B, Sim J, Kingstone T, Baker S, Waterfield J, Bartlam B (2018). Saturation in qualitative research: exploring its conceptualization and operationalization. Qual Quant.

[18] Borkan J, Crabtree B, Miller W (1999). Doing qualitative research.

[19] Charmaz K (2006). Constructing grounded theory: a practical guide through qualitative analysis: sage.

[20] Davies D, Dodd J (2002). Qualitative research and the question of rigor. Qual Health Res.

[21] Hinami K, Whelan CT, Wolosin RJ, Miller JA, Wetterneck TB (2012). Worklife and satisfaction of hospitalists: toward flourishing careers. J Gen Intern Med.

[22] Buerhaus PI, Donelan K, Ulrich BT, Norman L, Dittus R (2005). Is the shortage of hospital registered nurses getting better or worse? Findings from two recent national surveys of RNs. Nurs Econ.

[23] Wagner A, Rieger MA, Manser T, Sturm H, Hardt J, Martus P (2019). Healthcare professionals’ perspectives on working conditions, leadership, and safety climate: a cross-sectional study. BMC Health Serv Res.

[24] Singer S, Lin S, Falwell A, Gaba D, Baker L (2009). Relationship of safety climate and safety performance in hospitals. Health Serv Res.

[25] Jackson JL, Kroenke K, Chamberlin J (1999). Effects of physician awareness of symptom-related expectations and mental disorders. A controlled trial. Arch Fam Med.

[26] Döpp CM, Graff MJ, Teerenstra S, Nijhuis-van der Sanden MW, MGO R, Vernooij-Dassen MJ (2013). Effectiveness of a multifaceted implementation strategy on physicians’ referral behavior to an evidence-based psychosocial intervention in dementia: a cluster randomized controlled trial. BMC Fam Pract.

[27] Sinnenberg LE, Wanner KJ, Perrone J, Barg FK, Rhodes KV, Meisel ZF (2017). What factors affect physicians’ decisions to prescribe opioids in emergency departments?. MDM Policy Pract.

[28] Bot AG, Bekkers S, Arnstein PM, Smith RM, Ring D (2014). Opioid use after fracture surgery correlates with pain intensity and satisfaction with pain relief. Clin Orthop Relat Res.

[29] Nota SP, Spit SA, Voskuyl T, Bot AG, Hageman MG, Ring D (2015). Opioid use, satisfaction, and pain intensity after orthopedic surgery. Psychosomatics..

[30] Maher DP, Wong W, Woo P, Padilla C, Zhang X, Shamloo B (2015). Perioperative factors associated with HCAHPS responses of 2,758 surgical patients. Pain Med.

[31] Center for Medicare and Medicaid. Medicare Program: Hospital Outpatient Prospective Payment and Ambulatory Surgical Center Payment Systems and Quality Reporting Programs; Organ Procurement Organization Reporting and Communication; Transplant Outcome Measures and Documentation Requirements; Electronic Health Record (EHR) Incentive Programs; Payment to Nonexcepted Off-Campus Provider-Based Department of a Hospital; Hospital Value-Based Purchasing (VBP) Program; Establishment of Payment Rates Under the Medicare Physician Fee Schedule for Nonexcepted Items and Services Furnished by an Off-Campus Provider-Based Department of a Hospital 2016 [cited 2018 November 5]. Available from: https://www.federalregister.gov/documents/2016/11/14/2016-26515/medicare-program-hospital-outpatient-prospective-payment-and-ambulatory-surgical-center-payment. 27906530

[32] Epstein RM, Street RL. The values and value of patient-centered care. Ann Fam Med. 2011. 10.1370/afm.1239 PMC3056855 21403134

[33] Calcaterra SL, Yamashita TE, Min S-J, Keniston A, Frank JW, Binswanger IA (2016). Opioid prescribing at hospital discharge contributes to chronic opioid use. J Gen Intern Med.

[34] Donroe JH, Holt SR, Tetrault JM (2016). Caring for patients with opioid use disorder in the hospital. CMAJ.

[35] Chou R, Gordon DB, de Leon-Casasola OA, Rosenberg JM, Bickler S, Brennan T (2016). Management of Postoperative Pain: a clinical practice guideline from the American pain society, the American Society of Regional Anesthesia and Pain Medicine, and the American Society of Anesthesiologists' committee on regional anesthesia, executive committee, and administrative council. J Pain.

[36] Yajnik M, Hill JN, Hunter OO, Howard SK, Kim TE, Harrison TK (2019). Patient education and engagement in postoperative pain management decreases opioid use following knee replacement surgery. Patient Educ Couns.

[37] Balagué F, Mannion AF, Pellisé F, Cedraschi C (2012). Non-specific low back pain. Lancet.

[38] Burgess DJ (2010). Are providers more likely to contribute to healthcare disparities under high levels of cognitive load? How features of the healthcare setting may lead to biases in medical decision making. Med Decis Mak.

[39] Al Achkar M, Grannis S, Revere D, MacKie P, Howard M, Gupta S (2018). The effects of state rules on opioid prescribing in Indiana. BMC Health Serv Res.

[40] Dave DM, Grecu AM, Saffer H (2017). Mandatory access prescription drug monitoring programs and prescription drug abuse. Natl Bur Econ Res.

[41] Wen H, Hockenberry JM, Jeng PJ, Bao Y (2019). Prescription drug monitoring program mandates: impact on opioid prescribing and related hospital use. Health Aff.

