# Supplementary material for: Clinical perspectives on hospitals’ role in the opioid epidemic
Source: BMC Health Serv Res. 2020 Jun 8;20:521. doi: 10.1186/s12913-020-05390-4 (PMC7281936; doi:10.1186/s12913-020-05390-4)
Supplement: Supplementary file 1 — Additional file 1. The COREQ checklist by Tong, Sainsbury and Craig (2007) completed for the study: Clinical perspectives on hospitals’ role in the opioid epidemic. [file 12913_2020_5390_MOESM1_ESM.doc]

The COREQ checklist by Tong, Sainsbury and Craig (2007) completed for the study: Clinical perspectives on hospitals’ role in the opioid epidemic.

| ***Domain 1: Research team and reflexivity*** |
| --- |
| Personal characteristics |
| 1. It is reported that the first author conducted the interviews. |
| 1. The authors’ degrees are not reported in the main body of the manuscript. The first author holds an MD and PhD degrees. The second author holds a JD and PhD degrees. The third and fourth authors hold an MD degree. The fifth author holds a PhD degree. |
| 1. The authors’ occupations are not reported in the manuscript. When conducting this project and writing the paper the first and second authors were assistant professors with research being their predominant responsibility. The third and the fourth authors are active practicing clinicians. The fifth author is an associate professor and a researcher. |
| 1. The authors’ gender is not reported in the manuscript. The first, second, and fourth authors are females. The third and fifth authors are males. |
| 1. All authors have extensive training and experience in performing qualitative research. It is mentioned that the interviewer, i.e. the first author had extensive experience of interviewing at the time of data collection. The first, second, and fifth authors have an in-depth knowledge of health services research. The third and fourth authors have an in-depth knowledge of clinical practice. |
| Relationship with participants |
| 1. The study participants had no prior contact with the interviewer and other authors. |
| 1. All study participants were informed at all interviews about the purpose of the research. |
| 1. Interviewer characteristics were not reported to study participants. |
| ***Domain 2: Study design*** |
| Theoretical framework |
| 1. It is reported that data were analyzed with a modified version of the thematic analysis approach. |
| Participant selection |
| 1. It is reported that participants were recruited using purposive sampling approach. |
| 1. It is reported that potential participants were approached through e-mail communications and referrals. It is also reported that interested participants contacted the first author via e-mail to schedule an interview. All interviews were conducted in-person (see description of data collection on pp. 4-5). |
| 1. It is reported that the sample consists of 30 individuals. |
| 1. The number of eligible participants, the number who agreed to participate and the attrition rate is reported. |
| Setting |
| 1. The interviews took place either at the first author’s office located on the University campus or at the participant’s offices located in the hospitals. These aspects are not mentioned in the manuscript. |
| 1. Nobody besides the interviewer and a study participant was present during the interviews. |
| 1. Important characteristics of the sample are reported both in the Results section and in the Table 1. |
| Data collection |
| 1. It is reported that interview questions were pilot-tested and that the interviewer was supportive and asked follow-up questions in order to help the respondent to elucidate his/her answers. |
| 1. We did not carry out repeat interviews with our study participants. |
| 1. It is reported that the interviews were audio-recorded. |
| 1. Field notes were not taken during or after the interviews. This is not mentioned. |
| 1. It is reported that the interviews lasted approximately 45 minutes. |
| 1. It is reported that we ensured data saturation in our analysis, such that no new themes were emerging from the interviews. |
| 1. Transcripts were not returned to participants. This is not mentioned. |
| *Domain 3: Analysis and findings* |
| Data analysis |
| 1. It is reported that the first and second author coded the data. |
| 1. The steps by which interview data were coded, is reported. |
| 1. It is reported that the themes were derived from data. |
| 1. It is reported that Dedoose qualitative analysis software was used throughout the analysis process. |
| 1. Participants did not provide feedback on findings. This is not mentioned. |
| Reporting |
| 1. Participants’ quotations are presented to illustrate categories and for each quotation the respondent’s number is reported. |
| 1. There is consistency between the data presented and the findings. |
| 1. The major themes are clearly presented in the Results and Discussion sections. |
| 1. The minor themes and description of diverse cases are not presented due to the space limitations. |
