# Supplementary material for: Clinical perspectives on hospitals’ role in the opioid epidemic
Source: BMC Health Serv Res. 2020 Jun 8;20:521. doi: 10.1186/s12913-020-05390-4 (PMC7281936; doi:10.1186/s12913-020-05390-4)
Supplement: Supplementary file 2 — Additional file 2. Clinicians Interview Guide. [file 12913_2020_5390_MOESM2_ESM.docx]

**Clinicians Interview Guide**

**Introduction**

I am working on a project funded by the Indiana University to better understand existing pain care practices for patients in the hospital. The findings from this study will help clinicians to deliver safe and patient-centered care. As a clinician who is taking care of hospitalized patients, your perspective is very valuable to our study.

The focus of this interview is to gather your thoughts about patient experiences and pain care at your hospital. There are three parts to our discussion today.

• General information about your position (role; location of your practice; etc.)

• Concept of patient-centered care, including patients with pain

• Concept of safe care, including patients with pain

Do you have any questions before we begin our conversation?

Before we get started, I wanted to ask if it would be all right if we record this discussion. We only do this because we can’t write everything down. The recordings will be treated as confidential and only shared with the research team. If at any point you would like us to turn off the recorder, just let me know.

If you don’t have any questions, please review and sign this informed consent form. This informed consent form contains detailed information about this study, as we’ve discussed above. If you agree to be interviewed, you will be one of 30 individuals who will be participating in this research project. During the interview that will last approximately 45 minutes, you will share your perceptions on patient-centered care, patient safety, and the relationship between these two constructs. You are free to decline to answer any question and to end your participation at any point. This consent form indicates that you are willing to participate in the study. There are risks involved in all research studies. This study includes only minimal risks. For example, you may become uncomfortable when answering some questions. You can decline to answer any question at any time without any negative consequences. Your participation in this study cannot guarantee any direct benefits. However, your participation may help policy makers, health care administrators, and clinicians in their efforts to improve care while also curbing the national opioid epidemic.

General information

Please describe your role in the hospital (prompts: how long have you been with this hospital; did you work at other IU facilities prior to this role; do you practice in other IU facilities; what type of patients do you mostly see; are you a full-time or part-time employer; are you involved in research)

__________________________________________________________________________________________________________________________________________________________________________________________________________________________________________

Patient-centeredness

What is your understanding of a concept of patient-centered care? [Probs: Do you think about patient-centeredness when you are treating patients? If yes, how? If not, why?]

__________________________________________________________________________________________________________________________________________________________________________________________________________________________________________

How do you think about patients’ preferences and values when you are treating them? [Probs: Do you ask for patient’s preference prior to choosing a treatment plan? If yes, what do you ask? If no, why you don’t ask?]

__________________________________________________________________________________________________________________________________________________________________________________________________________________________________________

How do you think about patients’ experiences while there are in the hospital? [Probs: Are there explicit things that you do to make sure that this patient is having a good experience/is satisfied? If yes, what are these things? If not, why you don’t consider patient experiences? Do you feel that it is beyond your job description to take care of patient’s experiences/satisfaction while they are in the hospital?]

__________________________________________________________________________________________________________________________________________________________________________________________________________________________________________

Patient-centeredness & pain care/opioids

Earlier in our discussion, we talked about a concept of patient-centered care. How do you apply this concept to patients with pain? [Probs: Do you see that patients have different preferences for their pain care? If yes, what are they? If not, why do you think patients are asking for the same pain care?]

__________________________________________________________________________________________________________________________________________________________________________________________________________________________________________

Are there differences between non-surgical and surgical patients in terms of preferences for pain care? If yes, what are they? If not, why do you think these patients have similar preferences?

__________________________________________________________________________________________________________________________________________________________________________________________________________________________________________

Do you think that concerns about patient experience affects your decisions around pain care? If yes, how? If not, why? Can you give an example? Are there differences between surgical and non-surgical patients? [Additional probs: How do you factor in patient’s preferences for a particular pain medication, such as opioids, in your clinical decision-making process? Is it easier accomplished for some patients versus others? Can you give an example?]

__________________________________________________________________________________________________________________________________________________________________________________________________________________________________________

Patient satisfaction surveys

Tell me please your experiences with patient satisfaction surveys [Probs: Do they affect your decision-making? If yes, how? If not, why?]

__________________________________________________________________________________________________________________________________________________________________________________________________________________________________________

Do you see patient satisfaction surveys affecting other clinicians/your clinical team decision-making processes? If yes, how? If not, why?

__________________________________________________________________________________________________________________________________________________________________________________________________________________________________________

Several hospitals use satisfaction surveys to assess the quality of care. What is your opinion about this approach [Probs: Are you supportive of this approach? If yes, how? If not, why?]

__________________________________________________________________________________________________________________________________________________________________________________________________________________________________________

Patient Safety

How do you think about patient safety when you are treating patients? [Probs: Do you consider patient safety when you are choosing a treatment plan for a particular patient? If yes, how? If not, why?]

__________________________________________________________________________________________________________________________________________________________________________________________________________________________________________

How do you think about risks/adverse events when you are choosing a treatment plan for a particular patient? [Probs: Are there certain things/events you are trying to avoid? Are these things different for surgical versus non-surgical patients?]

__________________________________________________________________________________________________________________________________________________________________________________________________________________________________________

Are you more concerned about certain short-term or long-term risks of treatment while patient is in the hospital? Why? Do you think differently about short versus long-term risks for surgical versus non-surgical patients?

__________________________________________________________________________________________________________________________________________________________________________________________________________________________________________

Is it your job to think about patient safety issues? If yes, how? If not, why? [Additional probs: how do you work with your team to address potential patient safety issues?]

__________________________________________________________________________________________________________________________________________________________________________________________________________________________________________

Patient Safety & pain care/opioids

Please describe your approach to pain care for hospitalized patients. [Probes: How do you decide who will be given opioids? What objective and subjective information do you take into consideration when making a decision to prescribe opioids versus other pain medications? Can you give an example of a patient who would be a good candidate to receive opioids?]

__________________________________________________________________________________________________________________________________________________________________________________________________________________________________________

Let’s talk about pain care and patient safety. How do you approach patient safety when you manage patient with pain? [Probs: Do you worry about ADEs, such as sedation/OD/SUD when you prescribe opioids? If yes, how? If not, why?]

__________________________________________________________________________________________________________________________________________________________________________________________________________________________________________

HCAHPS/P.S. surveys/pain care

Existing patient satisfaction surveys ask patients about their satisfaction with pain care provided while they were in the hospital. What is your opinion about these scores? [Probs: Do you feel that these scores are affecting your decision-making process? If yes, how? If not, why?]

Patient Safety & Pt satisfaction

Let’s talk again about patient satisfaction. How patient safety aligns with patient satisfaction? [Probs: Do you think it is feasible to provide care that is safe and leads to high patient satisfaction? If yes, how? If not, why?]

__________________________________________________________________________________________________________________________________________________________________________________________________________________________________________

Is there anything else we should have asked you about patient-centeredness, patient experiences, patient safety and pain care that we did not? Anything else we should know? [Probs: What are the biggest challenges in providing safe and patient-centered care? What is an ideal pain care approach to hospitalized patients?]

__________________________________________________________________________________________________________________________________________________________________________________________________________________________________________

Do you think that the hospital is contributing to the opioid epidemic? (Explain your answer):

______________________________________________________________________________________________________________________________________________________________________________________________________________________________________________________________

If the participants answered yes to the first question, then ask: What can hospitals do to help address the opioid epidemic?

______________________________________________________________________________________________________________________________________________________________________________________________________________________________________________________________

**Closing**

Thank you again for taking the time to speak with us. Your first-hand perspective is very valuable to our research. We have learned a lot from this discussion.
